# Distribution and Quantification of Infectious and Parasitic Agents in Managed Honeybees in Central Italy, the Republic of Kosovo, and Albania

**DOI:** 10.3390/microorganisms14010219

**Published:** 2026-01-17

**Authors:** Franca Rossi, Martina Iannitto, Beqe Hulaj, Luciano Ricchiuti, Ani Vodica, Patrizia Tucci, Franco Mutinelli, Anna Granato

**Affiliations:** 1 Istituto Zooprofilattico Sperimentale dell’Abruzzo e del Molise “G. Caporale”, Via Campo Boario, 64100 Teramo, Italy; m.iannitto@izs.it (M.I.); l.ricchiuti@izs.it (L.R.); p.tucci@izs.it (P.T.); 2 Veterinary Laboratory, Food and Veterinary Agency, Ministry of Agriculture Forestry and Rural Development, Zona Industriale, Peja St. nr 241, 10000 Prishtine, Kosovo; bhulaj@yahoo.com; 3 Animal Health Department Food Safety and Veterinary Institute Aleksander Moisiu Nr 82, 1001 Tirana, Albania; ani.vodica@isuv.gov.al; 4 NRL for Honey Bee Health, Istituto Zooprofilattico Sperimentale delle Venezie, Viale dell’Università 10, 35020 Legnaro, Italy; fmutinelli@izsvenezie.it (F.M.); agranato@izsvenezie.it (A.G.)

**Keywords:** infectious and parasitic agents, managed honeybees, regional screening, distribution, contamination levels, co-occurrence

## Abstract

This study aimed to determine the presence of relevant infectious and parasitic agents (IPAs) in managed honeybees from Central Italy and the Republic of Kosovo and Albania to assess the overall health status of local apiaries by determining the contamination levels and co-occurrence. Therefore, pathogens and parasites such as *Paenibacillus larvae*, *Melissococcus plutonius*, *Vairimorpha apis*, *V*. *ceranae*, the acute bee paralysis virus (ABPV), black queen cell virus (BQCV), chronic bee paralysis virus (CBPV), deformed wing virus variants DWV-A and DWV-B, and the parasitoid flies *Megaselia scalaris* and *Senotainia tricuspis* were detected by quantitative polymerase chain reaction (qPCR) and reverse transcriptase qPCR (RT-qPCR) in clinically healthy adult honeybees collected from 187 apiaries in the Abruzzo and Molise regions of Central Italy, 206 apiaries in the Republic of Kosovo in 2022 and 2023 and 18 apiaries in Albania in 2022. The percentages of positive samples and contamination for *V. ceranae*, *P. larvae* and DWV-B were significantly higher in the Republic of Kosovo and Albania, while the percentages of samples positive for *M. plutonius*, CBPV, DWV-A, and the parasitoid flies were higher in Central Italy. Additionally, *P. larvae* and some viruses showed significantly different occurrence rates between the two years in Italy and the Republic of Kosovo. The co-occurrence of IPAs also differed between the two geographic areas. Their varying distribution could depend on epidemiological dynamics, climatic factors, and management practices specific to each country, whose relative impact should be defined to guide targeted interventions to reduce honeybee mortality.

## 1. Introduction

Apiculture is a source of income for the production of honey, beeswax, royal jelly, and propolis and an environment friendly farming practice for its relevant role in crop pollination and biodiversity conservation [1]. The western honeybee *Apis mellifera* is the pollinator with the highest frequency of floral visits and number of visited plant species worldwide [2]. Unfortunately, both managed and wild populations of *A*. *mellifera* are declining for causes such as habitat loss, climate change, irregular feeding, inappropriate management, exposure to pesticides and diseases [3,4,5]. The most recent officially released data for honeybee colony losses in European countries regard Switzerland and Liechtenstein and report winter colony losses ranging between 15.3% and 28.9% in different provinces and an increase in 2024/2025 compared to 2023/2024 in all locations (https://wildbeimwild.com/en/winter-losses-in-bee-colonies-2024-2025-analysis-and-risk-factors/, accessed on 30 December 2025).

Honeybee diseases are caused by infectious and parasitic agents (IPAs), which can lead to colony loss either directly, due to their high pathogenicity, or indirectly by weakening the colony and making it more susceptible to the harmful effects of concurrent infections, diseases, and environmental stresses [6]. The IPAs that most frequently affect honeybee health beyond the *Varroa destructor* mite, which is endemic in Europe and is managed by beekeepers with annual treatments [7], are of bacterial, fungal, viral and parasitic nature. These include the bacteria *Paenibacillus larvae* and *Melissococcus plutonius* which are both able to persist in the hives and cause the destructive and highly contagious diseases American and European foulbrood, respectively; the microsporidia *Vairimorpha apis* and *V. ceranae*, which are intracellular parasites that cause an intestinal infection called nosemosis and considerably impair colony strength; the honeybee viruses acute bee paralysis virus (ABPV), black queen cell virus (BQCV), chronic bee paralysis virus (CBPV), and deformed wing virus variants DWV-A and DWV-B, which cause diverse symptoms and all can lead to honeybee death if present at high levels; and the parasitoid flies *Megaselia scalaris* and *Senotainia tricuspis* whose larvae, deposited on the honeybee body, feed on the host, leading it to death [8,9,10,11].

According to the European legislation, only certain honeybee diseases are subject to surveillance and are assigned to different categories of required prevention measures. Specifically, *Varroa destructor* is assigned to the category C, i.e., diseases for which measures are needed to prevent the spread to parts of the Union that are officially disease-free or have eradication programs for the disease. *Tropilaelaps* spp., *Aethina tumida*, and American foulbrood are assigned to the category D, i.e., diseases that require measures to prevent their spread after entry into the Union and for movement between Member States. All these IPAs are also included in category E, which requires their surveillance within the Union. Eradication is not mandatory for any honeybee disease agent listed above under EU regulation, but it can be adopted by Member States in emergency situations [12]. All other diseases caused by honeybee IPAs are not included in national surveillance plans and are often diagnosed only on a voluntary basis. Nevertheless, these agents can significantly impact the survival of honeybee colonies [11,13,14,15,16,17,18,19], to the extent that their proactive control was requested more than a decade ago by the European Commission to prevent the decline of pollinating honeybee populations [20].

Although authoritative veterinary guidelines still recommend diagnosing honeybee disease agents only after clinical symptoms appear [8], proactive screening of IPAs in clinically healthy hives could help identify the risk of disease development and allow timely intervention to limit or prevent its manifestation and spread by applying appropriate countermeasures [21]. Therefore, the aim of this study was to provide an overview of the IPAs threatening managed honeybees in the Abruzzo and Molise regions of Central Italy, the Republic of Kosovo, and four provinces of Albania in years 2022 and 2023. Abruzzo and Molise are located at nearly the same latitudes as the Republic of Kosovo and Albania but differ in distance from the coast and apiary density. In 2023, Abruzzo and Molise (Italy) had 84,576 hives, a number similar to that in 2022 [22], while the Republic of Kosovo had 208,898 hives [23], and Albania had 519,000 hives (https://www.instat.gov.al/en/themes/agriculture-and-fishery/livestock/#tab2/, accessed on 10 June 2025). Additionally, in the Republic of Kosovo, but not in Italy, the government provides financial compensation to beekeepers for each hive lost to AFB and EFB [24]. The pathogens considered were the bacteria *P. larvae* and *M. plutonius*, the microsporidia *V*. *ceranae* and *V*. *apis*, the viruses acute bee paralysis virus (ABPV), black queen cell virus (BQCV), chronic bee paralysis virus (CBPV), deformed wing paralysis virus variants DWV-A and DWV-B, and the parasitoid flies *M. scalaris* and *S. tricuspis*, which were detected in the geographic areas examined in a previous study [11]. Adult honeybees were chosen for IPA detection because their suitability for the surveillance of all the pathogens and parasites considered was demonstrated [8,9,11,25,26].

## 2. Materials and Methods

### 2.1. Sample Collection and Storage

This study addressed the diagnosis and definition of IPA infection levels affecting honeybees during the spring and summer of 2022 and 2023 in the Abruzzo and Molise regions of Central Italy, the Republic of Kosovo, and four provinces of Albania. It was conducted jointly by the responsible veterinary surveillance institutes in these countries. Sampling was conducted from May to early July in all provinces of the Abruzzo and Molise regions, Italy, and in five provinces of the Republic of Kosovo. In Albania, samples were collected only in 2022 in four provinces. The apiaries were selected on the basis of even distribution in the geographical regions examined and sampling possibility depended on the interest in participating to the survey of the beekeepers included in lists of the veterinary institutions that conducted the research. There was not a criterion for the hive choice but only the requirement of being located in the middle of the apiary and of appearing healthy.

**Figure 1:**
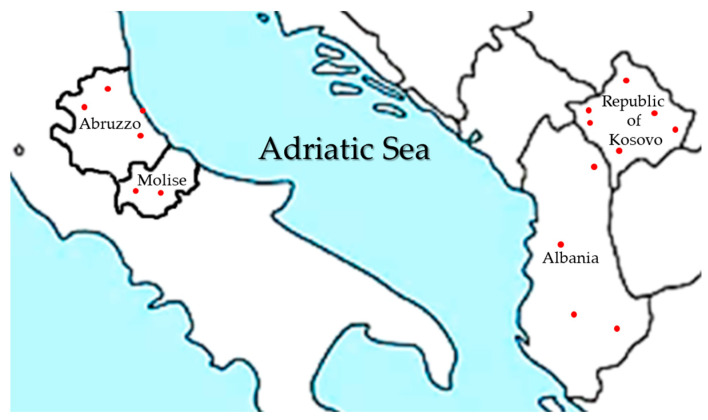
Geographic position of Abruzzo and Molise Italian regions, Republic of Kosovo and Albania and capitals of the provinces where sampling was conducted (red dots).

The geographic position of the regions and provinces in which sampling was conducted is shown in Figure 1.

In 2022, 131 honeybee samples were collected in the regions of Abruzzo and Molise (Italy), 140 in the Republic of Kosovo, and 18 in Albania. In 2023, 56 samples were collected in Abruzzo and Molise, and 66 in the Republic of Kosovo and sampling was limited to one apiary representative for nearby sites that had shown identical pathogen presence/absence profiles in 2022.

Approximately 40 honeybees were collected from the top of each hive by dragging a Falcon tube (Merck, KGaA, Darmstadt, Germany) along the frame edges while using a bee smoker. From each apiary one hive was sampled. This was chosen among those without signs of disease and located in the middle of the apiary. The insects were immediately refrigerated and transported within 48 h to the laboratory, where they were divided into aliquots for DNA and RNA extraction. The aliquots not used promptly for analysis were immediately frozen at −80 °C. Samples collected in the Republic of Kosovo and Albania were placed in approximately five volumes of RNAlater solution per g of honeybees, namely 16 mL of storage solution for 40 honeybees (Thermo Fisher Scientific, Rodano, Italy) according to the manufacturer’s instructions before being transported at room temperature to the Campobasso branch of the Istituto Zooprofilattico Sperimentale dell’Abruzzo e del Molise (IZSAM), Italy, for molecular analyses.

### 2.2. Nucleic Acid Extraction

Samples examined for all IPAs except parasitoid flies consisted of two adult honeybees from one hive per apiary, rather than pooled samples, as higher sensitivity had previously been observed for non-pooled samples [27,28]. DNA was extracted from two bees in the same tube using the “small-scale” method previously described [11] to detect bacteria and microsporidia, with the modification that 400 μL of T1 buffer (Carlo Erba, Cornaredo, MI, Italy), instead of 200 μL, was added to the sample before mechanical lysis. For the detection of *M. scalaris* and *S. tricuspis*, the large-scale method previously described [11] was applied to 20 bees. A DNA extraction control consisting of homogenized *T. molitor*, kindly provided by Dr Sauro Simoni (CREA—Consiglio per la Ricerca in Agricoltura—Difesa e Certificazione, Firenze, Italy), was added to the sample before extraction as described by Rossi et al. [11]. RNA extraction was carried out from two honeybees as previously described, using *T. molitor* as the RNA extraction control [10]. The bees used for nucleic acid extraction were randomly picked among those sampled from a single hive and were used entirely and not cleaned from pollen to avoid the loss of IPAs located on the surface of the bee body. The two-bee or 20-bee aliquots were weighed to refer the results of pathogen or parasite contamination level to one gram of insects and the whole bodies were homogenized in the same tube for DNA extraction.

### 2.3. Quantitative PCR and RT-qPCR

A quantitative polymerase chain reaction (qPCR) or reverse transcriptase qPCR (RT-qPCR) was performed to determine the abundance of each pathogen in the samples using the extracted DNA or RNA, following previously described assays [9,10,11,29]. The *P. larvae*-specific qPCR protocol was based on Rossi et al. [30], with the modification of replacing Sybr Green with the Cy5-TGTAAGACCGGGATAACTT-MGBEQ probe at a 0.2 µM concentration. In the qPCR, DNA extracted from the bacterial type strain *P. larvae* ATCC 9545 was used as the positive control for *P. larvae*, while recombinant plasmids containing the pathogen-specific DNA fragment, supplied by GenScript Biotech (Rijswijk, Netherlands), were used for *M. plutonius*, *V. apis*, *V. ceranae*, *M. scalaris*, and *S. tricuspis* [9,11,29,30]. For viral pathogens, RT-qPCR positive controls consisted of synthetic RNA fragments corresponding to the specific target region of the investigated viruses (GenScript Biotech, Rijswijk, Netherlands) [22]. Negative process controls (400 μL of nuclease-free water in place of the bee sample) and negative amplification controls (nuclease-free water in place of DNA or RNA) were included in each extraction and amplification run. The qPCR and RT-qPCR reactions for each pathogen were performed using a QuantStudio 5 instrument (Thermo Fisher Scientific, Rodano, Italy) as previously described [9,10,11,30].

The quantification of viral pathogens in honeybee samples was performed as described by Rossi et al. [10], while *M. plutonius*, *V. apis*, *V. ceranae*, and *P. larvae* were quantified using standard curves constructed in this study, with their linearity range shown in Figure S1. The copy number of IPAs in samples with Ct values outside the linearity range was estimated by comparison with honeybee samples spiked in triplicate with copy numbers of pathogen-specific recombinant plasmids or cells external to the linearity range. For *M. scalaris* and *S. tricuspis*, calibration curves were not constructed because their levels in adult honeybees were previously found to be close to or below the limit of detection (LOD) [11]. Therefore, approximate quantification was achieved by comparison with honeybee samples spiked in triplicate with 2 and 3 log copy numbers of the fly-specific recombinant plasmids. For *M. plutonius* and the microsporidia, the standards consisted of DNA extracts from honeybee samples spiked in triplicate with 10-fold serial dilutions from 1 log copies/g to 8 log copies/g of the pathogen-specific recombinant plasmids.

For *P. larvae*, DNA quantification standards with known colony forming units (CFU) were prepared from serial decimal dilutions of a *P. larvae* ATCC 9545 cell suspension counted on PLA medium and used to inoculate bees in triplicate with contamination levels ranging from 1 log CFU/g to 8 log CFU/g. of The *P. larvae* cell suspension was prepared by streaking a single colony of *P. larvae* ATCC 9545 on *Paenibacillus larvae* agar (PLA) [31], incubating at 37 °C for 2–5 days in a 9% CO_2_ atmosphere incubator, and resuspending the colonies grown on the entire plate in 2 mL of sterile saline.

The honeybee samples used for constructing all standard curves had previously been tested in triplicate by qPCR to verify absence of amplification in the IPA-specific tests.

### 2.4. Statistical Analyses

Principal Component Analysis (PCA), Pearson’s correlation analysis, and graphical representations were performed using the Past 4.17 software (https://www.nhm.uio.no/english/research/resources/past/, accessed on 12 November 2025). The data used in the PCA consisted of the ratios of samples positive in the qPCR or RT-qPCR tests to the total sample number for each IPA in each province. Student’s *t*-test was conducted with Microsoft Excel 2016 to evaluate the statistical distinctness of presence/absence data based on the detection by qPCR or RT-qPCR for each IPA between the two years in the same geographic area.

## 3. Results

According to the qPCR and RT-qPCR results, no samples were free of IPAs, and the number of co-occurring pathogens or parasitic agents ranged from two to ten in Italy in 2022, four to eight in Italy in 2023, six to seven in Albania in 2022, and three to eight in the Republic of Kosovo in both years. Table S1 shows the co-infection profiles observed by year and geographic area, while Table 1 shows the number of samples positive for each pathogen and parasite in the different provinces or districts over the two years. *V. apis* was not detected in any sample, and *M. plutonius*, *M. scalaris*, and *S. tricuspis* were not detected in Albania.

**Figure 2:**
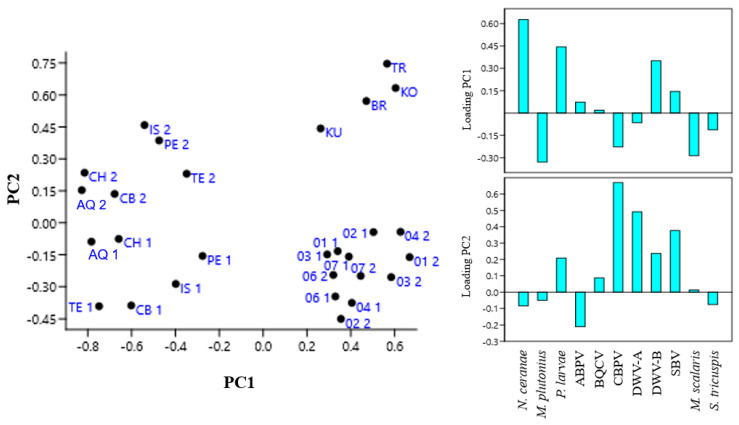
Principal component analysis (PCA) of samples obtained by using as variables the ratio of positive samples on total honeybee samples for each infectious and parasitic agent (IPA) in each province of Abruzzo and Molise regions (Italy) and of the Republic of Kosovo and Albania in 2022 (1) and 2023 (2). AQ, L’Aquila; CB, Campobasso; CH, Chieti; IS, Isernia; PE, Pescara; TE, Teramo; 01, Pristina; 02, Mitrovica; 03, Peja; 04, Prizren; 06, Gjilane; 07, Gjakova; BR, Berat; KU, Kukës; TR, Tiranë; KO, Korçë.

A principal component analysis (PCA) was conducted to evaluate if samples could be distinguished on the basis of IPA presence and was obtained by using as variables the ratios of positive samples to total samples for each pathogen in each province. In PCA the two first principal components PC1 and PC2 report the widest variance intervals for the variables used in the analysis, while the loading plot identifies the variables that mostly influence this variance. A high loading value for a certain variable on a PC indicates that its contribution to the sample variance is relevant. The results of this analysis are shown in Figure 2.

The PCA showed that the occurrence of *V. ceranae*, *P. larvae*, and DWV-B mainly influenced sample distribution on PC1, while CBPV, DWV-A, and SBV, in that order, were the main variables influencing sample distribution on PC2. Samples from the Abruzzo and Molise regions formed distinct clusters along PC2 according to the sampling year, suggesting that one or more variables influencing PC2 changed between the two sampling periods. Conversely, samples from the Republic of Kosovo collected in the two years were intermixed along PC1 and PC2, indicating an unchanged situation in the variables that most influence the PCs. Furthermore, samples from the Italian regions for 2022 and those from the Republic of Kosovo were not separated along PC2, indicating that they did not differ significantly for the variables with positive loading on this PC. Samples from Albania were separate from those collected in the Republic of Kosovo on PC2, while they were not separate on PC1, indicating that the variance in the presence of *V. ceranae*, *P. larvae*, and DWV-B did not differ between the two neighboring countries.

**Figure 3:**
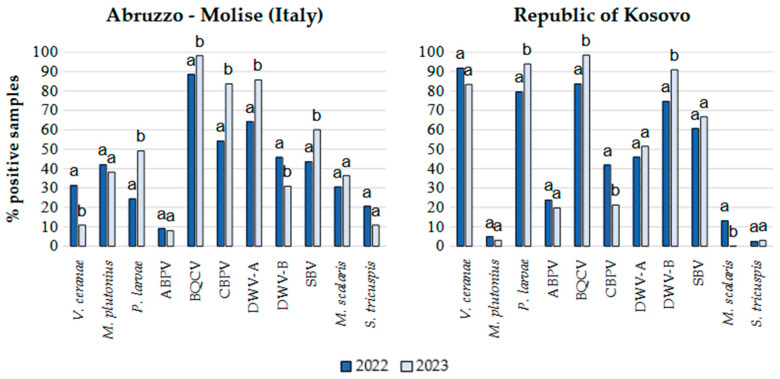
Percentages of apiaries positive for honeybee infectious and parasitic agents (IPAs) in 2022 and 2023 in the Abruzzo and Molise regions (Italy) and in the Republic of Kosovo. Different letters on the bars indicate significantly different infection or infestation rates between the two sampling periods (*p* < 0.05).

Since samples from the two geographic areas clustered closely in the PCA, the percentages of positive samples for each pathogen in the two years for the Italian regions and the Republic of Kosovo were determined from the total number of samples from each country (Figure 3). Samples from Albania were not included in Figure 3 due to their low number.

Based on Student’s *t* test, a significant increase in the number of samples positive for *P. larvae*, BQCV, CBPV, DWV-A, and SBV was observed in 2023 in the Italian regions, while the detection frequency for *V. ceranae* and DWV-B significantly decreased (Figure 2). In 2023 in the Republic of Kosovo, the number of samples positive for *P. larvae*, BQCV and DWV-B significantly increased, while those positive for CBPV and *M. scalaris* significantly decreased. *V. ceranae*, *P. larvae* and DWV-B were detected more frequently in the Republic of Kosovo in both years, whereas *M. plutonius* was more frequently detected in the Italian regions, with a much lower occurrence in the Republic of Kosovo. CBPV, DWV-A, and the parasitoid flies occurred more frequently in Italy, while DWV-B was more frequently detected in the Republic of Kosovo.

**Figure 4:**
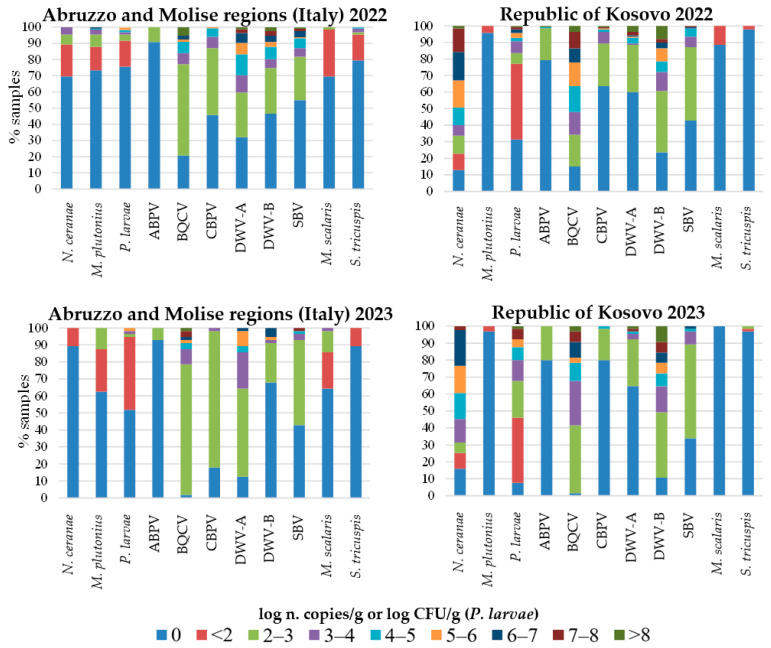
Quantification of honeybee infectious and parasitic agents (IPAs) determined by qPCR and RT-qPCR, and percentages of samples per range of pathogen loads in 2022 and 2023 in the Abruzzo and Molise regions (Italy) and in the Republic of Kosovo. For viruses, levels below 2 log n. copies/g were not considered, as they are below the limit of detection of the methods used [10].

**Figure 5:**
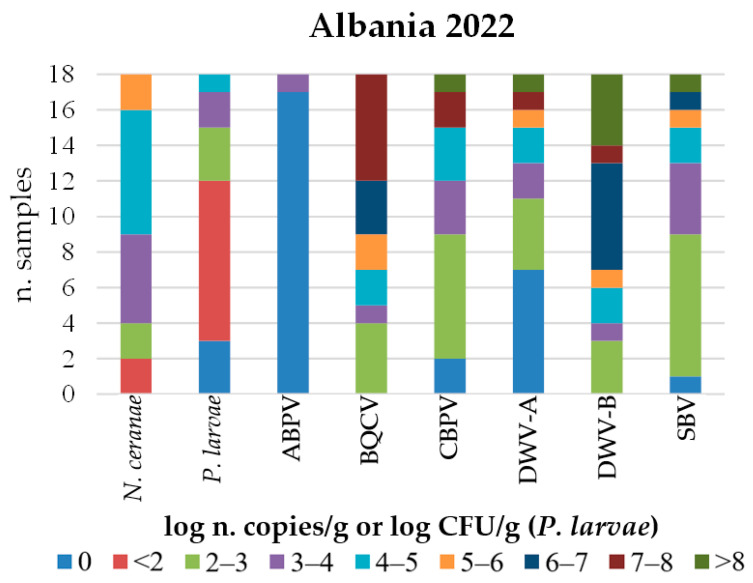
Distribution of honeybee samples collected in Albania in 2022 based on the quantification of each detected infectious and parasitic agent (IPA).

The quantification of IPAs, determined by qPCR and RT-qPCR in Italy and the Republic of Kosovo, is shown in Figure 4 along with the respective percentages of positive samples. Samples from Albania are presented separately, showing the number of samples per quantification level, but only for the IPAs detected in that country (Figure 5). These were not expressed in percentages given the small number of samples from that country and to provide a clearer graphic representation.

A striking difference between the geographic areas was observed in the abundance of *V. ceranae* and *P. larvae*. In some samples collected in the Republic of Kosovo, *V. ceranae* and *P. larvae* reached very high numbers ranging between 7 and 8 log CFU/g or log n. copies/g, respectively, in both years. In contrast, in the Italian regions, the *P. larvae* maximum number was 1 or 2 log CFU/g lower than in the Republic of Kosovo in both years and *N. ceranae* reached relatively low copy numbers of 3 to 4 log n. copies/g in 2022 and even lower in 2023.

*M. plutonius* reached levels between 6 and 7 log n. copies/g in a few Italian samples in 2022, while it decreased of up to 4 log n. copies/g in 2023. This pathogen was always below 2 log n. copies/g in samples from the Republic of Kosovo in both years.

The BQCV was present at very high viral loads (higher than 8 log n. copies/g) in some samples from both geographic areas and in both years, though in the Italian regions, the percentage of samples with low viral loads was higher. The DWV-A and DWV-B reached the highest viral loads, higher than 8 log n. copies/g, in samples from both geographic areas in 2022, while in 2023, the DWV-A and DWV-B decreased of at least 1 log n. copies/g in Italy. The percentage of samples with the highest levels of DWV-B was higher in the Republic of Kosovo in both years.

The percentage of samples positive for the parasitoid flies was higher in Italy in both years. However, in 2023, an increase in *S. tricuspis* abundance to above 2 log n. copies/g was observed in one sample from the Republic of Kosovo.

Although only a few samples from Albania were examined, some differences in IPA abundance were observed compared to the Republic of Kosovo, namely, a lower abundance of *V. ceranae* and *P. larvae*. In contrast, the abundance of CBPV and SBV was higher in Albania, with both reaching loads above 8 log n. copies/g. Furthermore, the proportion of DWV-B positive samples with viral loads above 4 log copies/g was higher in Albania (Figure 5).

**Figure 6:**
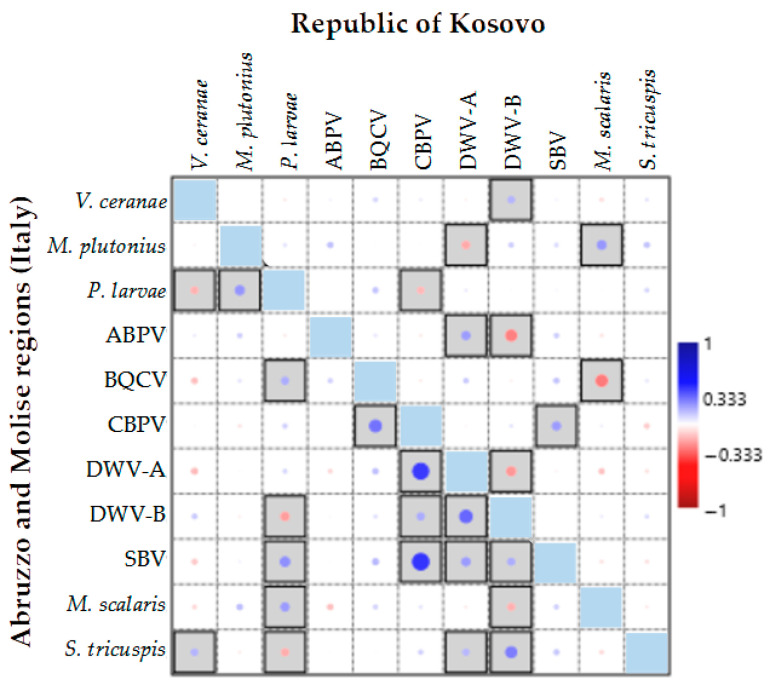
Pearson’s correlation analysis of the presence or absence of honeybee infectious and parasitic agents (IPAs) in the Abruzzo and Molise regions (Italy) and the Republic of Kosovo. Bordered boxes indicate significance at *p* < 0.05. Dot size represents the level of correlation.

A correlation analysis based on the presence or absence of the IPAs was conducted to identify possible co-infection trends in Italy and the Republic of Kosovo, as shown in the graphical representation in Figure 6.

The significant correlations observed between IPAs for the two geographic areas differed in most cases, except for the positive correlation between CBPV and SBV. Additionally, a positive correlation was observed between *M. scalaris* and *M. plutonius* as well as between the DWV-B and *V. ceranae*. While this correlation was not statistically significant for the Italian regions, it was significant for the Republic of Kosovo. The BQCV and *P. larvae* were positively correlated in both countries, but this correlation was not statistically significant for the Republic of Kosovo. Similarly, *P. larvae* and *M. plutonius* were negatively correlated in both countries, but the correlation was not significant for the Republic of Kosovo. In the Italian regions, significant positive correlations were observed for DWV-A with CBPV and DWV-B; for *S. tricuspis* with DWV-B, CPBV and BQCV; for SBV with *P. larvae* and both DWV variants; for *M. scalaris* and *P. larvae*; and for *S. tricuspis* and *V. ceranae*. Significant negative correlations (*p* < 0.05) were observed for *P. larvae* with *V. ceranae* and DWV-B and between DWV-B, and *M. scalaris*. For the Republic of Kosovo, a significant positive correlation was observed between DWV-A and ABPV, while ABPV was negatively correlated with DWV-B, BQCV and *M. scalaris*, CBPV was negatively correlated with *P. larvae*, and DWV-A was negatively correlated with DWV-B and *M. plutonius* (Figure 6).

## 4. Discussion

This study, which focused on detecting and quantifying multiple honeybee IPAs by qPCR and RT-qPCR in relatively small geographic areas, can be compared with a similar investigation conducted in 2016 and 2017 around Stuttgart, Germany, where the same techniques were applied to individual honeybees [32]. In that study, as found also in the present study (Table S1), among 1064 honeybee samples from colonies without clinical or pathological symptoms, none were free from pathogens. Similar results were obtained for the occurrence of BQCV, but the other viruses were less prevalent than in this study, except for ABPV in Italy and DWV-B in the Republic of Kosovo. The occurrence of *V. ceranae* was similar to that in the Republic of Kosovo and Albania. *M. plutonius* showed an intermediate occurrence rate between Italy and the Republic of Kosovo, and *P. larvae* had a similar detection rate as in Italy. The reported differences indicate that the spatio-temporal distribution of biotic risk for honeybees is not uniform, as also shown by the PCA conducted in this study, which revealed a distinction between the study periods. This observation suggests that periodic screening studies of local pathogens may be useful for early detection of disease threats, thereby improving honeybee health and productivity.

The high prevalence of the pathogens *P. larvae* and *V. ceranae* in the Republic of Kosovo could be a consequence of the hive compensation policy for honeybee colonies lost to AFB and EFB [33]. This policy likely results in a reduced control on honeybee infections by some beekeepers, facilitating the intense circulation of contagious agents, which is further aggravated by the high density of apiaries [23]. This undermines the efforts of conscientious beekeepers to maintain high levels of hive hygiene. The high prevalence of *V. ceranae* in the Republic of Kosovo is consistent with previous results from passive surveillance of weak honeybee colonies, which showed an infection rate of 100% [34]. Additionally, the results of recent interviews conducted with 200 beekeepers in the Republic of Kosovo confirm that the missed adoption of good beekeeping practices is perceived as one of the main factors affecting honeybee colony health and apiculture productivity in the country, along with climate change, land use, and pesticide exposure [35]. Therefore, it is possible that a change in legislation regarding AFB and EFB control in the Republic of Kosovo could promote the adoption of good beekeeping practices and, in general, contribute to a reduction in IPA loads. In the Republic of Kosovo, *M. plutonius* was infrequent and positively correlated with parasitoid flies, which also had low occurrence in that region. Therefore, the possible involvement of these or other insects in its spread in apiaries should be investigated. In a previous study [11], these flies were found to host microbial honeybee pathogens, so the possibility that they also propagate *M. plutonius* should be explored.

The results on the presence of honeybee viruses in the Republic of Kosovo partly agree with previous findings from a study of 13 apiaries, where the DWV and BQCV were most frequently detected, followed by the ABPV, SBV, and CBPV in that order [34]. This study showed a lower occurrence of the ABPV and a higher occurrence of the CBPV and SBV, but the observed differences likely result from examining clinically healthy honeybee colonies, unlike the previous study, which involved hives suspected of disease.

The relatively low frequency and levels of infection observed for *V. ceranae* and *P. larvae* in the Italian regions indicate effective control of these pathogens through adherence to good beekeeping practices. Moreover, using plant-based feed supplements containing natural antimicrobials might have effectively reduced *V. ceranae* infections but this possibility requires further investigations [36]. However, other studies conducted in Italy have reported a high frequency of detection of this parasite, suggesting the need to continue monitoring its presence [37,38].

Compared to a previous survey in the Abruzzo and Molise regions conducted in 2018 [39], the detection rate of *P. larvae* decreased in 2022 and then increased again in 2023, showing a fluctuating trend for this pathogen whose causes should be investigated. The most probable reason for its intermittent increase could be the varying levels of virulence among circulating strains. This should be confirmed by strain isolation and whole-genome sequencing (WGS) to identify virulence traits and sources of infection for sequence variants, in order to improve AFB prevention strategies.

The frequent occurrence of *M. plutonius* in Italy may reflect the epidemiological dynamics of this highly destructive brood pathogen, its dependence on climatic conditions, and the existence of transmission routes specific to the region. Previous studies have shown that the distribution of *M. plutonius* is influenced by climatic factors such as precipitation and temperature [40]. Climatic factors are most likely responsible for the higher incidence of parasitoid flies in Italy compared to the Republic of Kosovo, according to predictive models [41,42], and this could contribute to the spread of *M. plutonius* in Italy. The detection of *M. plutonius*, *M. scalaris*, and *S. tricuspis* in Albania might have been missed because of the small number of samples examined. Therefore, their presence in the country cannot be excluded, though it is likely low.

According to the most recent data on the prevalence of *M. plutonius* in Europe, this pathogen reached a 46% detection frequency in areas with high apiary density [43]. Additionally, a high prevalence of *M. plutonius* was reported in Michigan (US) in 2021 and 2022, where it varied seasonally with a peak in June, and different genetic variants were identified within the same beekeeping operation [44]. A high occurrence rate (32.4%) of different strains of this pathogen, with variability among sites, was also observed for *A. cerana* in Guangxi (China) and stingless bees in Brazil [45,46]. Therefore, this pathogen represents a significant threat to the productivity of the apicultural sector in different countries, whose causes should be defined and appropriately addressed. A four-gene multilocus sequence typing (MLST) scheme, used to discriminate the clonal complexes with a higher probability of causing EFB, and the detection of the plasmid-encoded *mtx*A gene for melissotoxin A production [47], could be applied to define the virulence of the genotypes infecting hives in Italy and to differentiate interventions based on the pathogenicity of the detected genotype.

All honeybee viruses, except the BQCV, were reported to be associated with the *V. destructor* mite, by experimental demonstration [34,48] or by statistical inference [49]. Consequently, their different distribution most likely reflects the prevalence of this parasite and its viral loads. The common source of infection can explain the multiple positive correlations in occurrence patterns found in this study for viruses in Italy, which indicate a high probability of co-infection. The impact of viral co-infections on overall hive health should be more clearly defined, taking into account both the identity and viral load of co-occurring viruses.

A different occurrence rate of the DWV-A and DWV-B was observed in this study between the two geographic areas examined, and the higher occurrence of DWV-A in Italy did not align with the general increasing trend of DWV-B over DWV-A reported in other countries [49,50,51]. The frequency of DWV-A detection was also slightly higher than that of DWV-B in the Veneto, Campania, and Sicily regions of Italy, according to recent studies [37,51]. Additionally, DWV-A was recently reported as the most abundant DWV variant in Michigan [52], indicating that specific variants of the DWV-A may outcompete DWV-B in different geographic locations. The use of a recently described amplicon sequencing strategy that links specific amplicon sequence variants (ASVs) to virulence [53] could experimentally verify this hypothesis.

For the Italian regions, detection results for the BQCV, DWV, and CBPV viruses were consistent with previous investigations conducted in other areas of Italy. A maximum infection rate of 84% for the CBPV in 2021–2023, similar to that found in this study in 2023, was previously reported [38,54]. The ABPV showed similar prevalence and low abundance in the Veneto and Piemonte regions [38,55], while in the present study its detection frequency was even lower, probably because the sampling period did not coincide with the peak prevalence of this pathogen, which occurs in late summer or autumn [56]. For the SBV, a study conducted in the Piemonte region during 2017–2020 is available for comparison and reported higher infection rates than those found in the present study [55].

A limitation of this study is the sampling of only one hive per apiary and the analysis of only two bees, except for parasitoid flies, from each hive. This may have led to an underestimation of the actual occurrence rates of IPAs. Future investigations will define the optimal number of hives to be sampled at the apiary level and number of samples per hive to exclude the presence of IPAs with certainty. Another limitation was not identifying IPAs with high genotype resolution to explain virulence and spread dynamics. In particular, DWV-A variants, *M. plutonius*, and *P. larvae* strains circulating in Italy should be characterized by WGS to identify the specific physiological traits that determine their selective success.

## 5. Conclusions

This investigation showed that the distribution of honeybee IPAs can vary even between neighboring countries and that disease threats to honeybee health and apiculture productivity require specific countermeasures based on those most frequently occurring in a given area. Regular local monitoring of IPAs and defining contamination trends can enable timely and focused interventions. Use of rapid, sensitive, specific and quantitative detection methods, even in the absence of clinical signs, greatly facilitate disease prevention and trend analysis.

This study also showed that the regulation of the beekeeping sector in the Republic of Kosovo needs substantial revision to encourage the adoption of good management practices aimed at reducing the occurrence and abundance of *P. larvae* and *V. ceranae*, which significantly compromise productivity and can spread to neighboring countries.

The high frequency of co-infection observed indicated that the determination of single pathogens may be not sufficient to define honeybee colony health, but rather the detection of multiple IPAs is needed.

Use of IPA variant discrimination by sequencing methods should be implemented to explain distribution and epidemiological trends of virulent biotypes.

## Figures and Tables

**Table 1:** Number of positive samples for the honeybee infectious and parasitic agents (IPAs) investigated in this study in the provinces of the Abruzzo and Molise regions (Italy) and in the districts of the Republic of Kosovo and Albania.

Province/ District	Year	N. samples	*V. apis*	*V. ceranae*	*M. plutonius*	*P. larvae*	ABPV	BQCV	CBPV	DWV-A	DWV-B	SBV	*M. scalaris*	*S. tricuspis*
	Abruzzo and Molise regions (Italy)													
	N. positive samples													
Campobasso	2022	26	0	7	10	6	3	21	11	15	9	11	7	4
	2023	10	0	2	5	3	1	10	9	8	8	3	4	2
Chieti	2022	26	0	6	6	1	1	21	16	22	17	12	8	11
	2023	10	0	1	3	3	0	10	8	10	4	6	6	1
Isernia	2022	18	0	8	8	6	3	16	10	9	11	7	5	2
	2023	7	0	0	4	7	0	7	6	7	3	5	2	0
L’Aquila	2022	25	0	4	18	15	2	23	16	15	4	17	14	4
	2023	10	0	1	4	1	0	9	7	9	5	8	5	2
Pescara	2022	17	0	11	3	1	2	16	11	12	12	7	2	4
	2023	8	0	0	1	6	0	8	8	6	5	5	2	0
Teramo	2022	19	0	5	9	2	4	19	3	7	2	3	4	2
	2023	11	0	2	4	7	3	11	8	11	8	6	1	1
	Republic of Kosovo													
	N. positive samples													
Gjakova	2022	45	0	41	2	32	07	36	17	27	38	28	6	2
	2023	19	0	14	0	16	30	18	3	11	16	12	0	0
Gjilane	2022	13	0	13	0	6	6	12	3	9	8	9	2	0
	2023	11	0	7	0	9	3	11	2	8	8	6	0	0
Mitrovica	2022	13	0	12	1	12	1	13	5	9	12	7	1	0
	2023	4	0	3	1	4	1	4	1	1	4	2	0	1
Peja	2022	29	0	24	1	18	4	23	13	16	26	16	5	0
	2023	9	0	8	1	9	3	9	1	6	7	6	0	0
Pristina	2022	14	0	14	0	7	5	13	8	7	11	10	2	0
	2023	12	0	12	0	12	2	12	3	7	10	8	0	0
Prizren	2022	26	0	21	2	21	5	24	6	12	21	11	0	1
	2023	11	0	10	0	11	1	10	3	7	10	9	0	1
	Albania													
	N. positive samples													
Kukës	2022	4	0	3	0	2	1	4	3	4	4	4	0	0
Berat	2022	4	0	4	0	3	0	4	4	3	4	4	0	0
Tiranë	2022	4	0	4	0	4	0	4	4	4	4	4	0	0
Korçë	2022	6	0	6	0	6	0	6	5	6	6	6	0	0

## Data Availability

Data supporting reported results are available upon request.

## Supplementary Materials

The following supporting information can be downloaded at: https://www.mdpi.com/article/10.3390/microorganisms14010219/s1. Figure S1: Calibration curves constructed in this study to quantify IPAs in honeybee samples with DNA extracted from honeybee samples negative for these pathogens and inoculated in three biological replicates with serial decimal dilutions of plasmid pUC57 containing the target region of *N. apis*, *N. ceranae*, or *M. plutonius* or with known numbers of *P. larvae* cells using previously reported qPCR methods [17,23,24]. Only the linearity ranges are shown. Table S1: IPAs co-infection profiles observed in honeybee samples from the Abruzzo and Molise regions (Italy), the Republic of Kosovo, and Albania in 2022 and 2023.

## Abbreviations

The following abbreviations are used in this manuscript:

ABPV	Acute bee paralysis virus
AFB	American foulbrood
ASV	Amplicon sequence variants
BQCV	Black queen cell virus
CBPV	Chronic bee paralysis virus
DWV-A	Deformed wings virus A
DWV-B	Deformed wings virus B
EFB	European foulbrood
IPA	Infectious or parasitic agent
LOD	Limit of detection
MLST	Multilocus sequence typing
PCA	Principal component analysis
PC1	Principal component 1
PC2	Principal component 2
qPCR	Quantitative PCR
RT-qPCR	Reverse transcriptase qPCR
SBV	Sacbrood virus
WGS	Whole-genome sequencing
