# Supplementary material for: Distribution and Quantification of Infectious and Parasitic Agents in Managed Honeybees in Central Italy, the Republic of Kosovo, and Albania
Source: Microorganisms. 2026 Jan 17;14(1):219. doi: 10.3390/microorganisms14010219 (PMC12843687; doi:10.3390/microorganisms14010219)
Supplement: Supplementary file 1 [file microorganisms-14-00219-s001.zip › Figure S1.pdf]

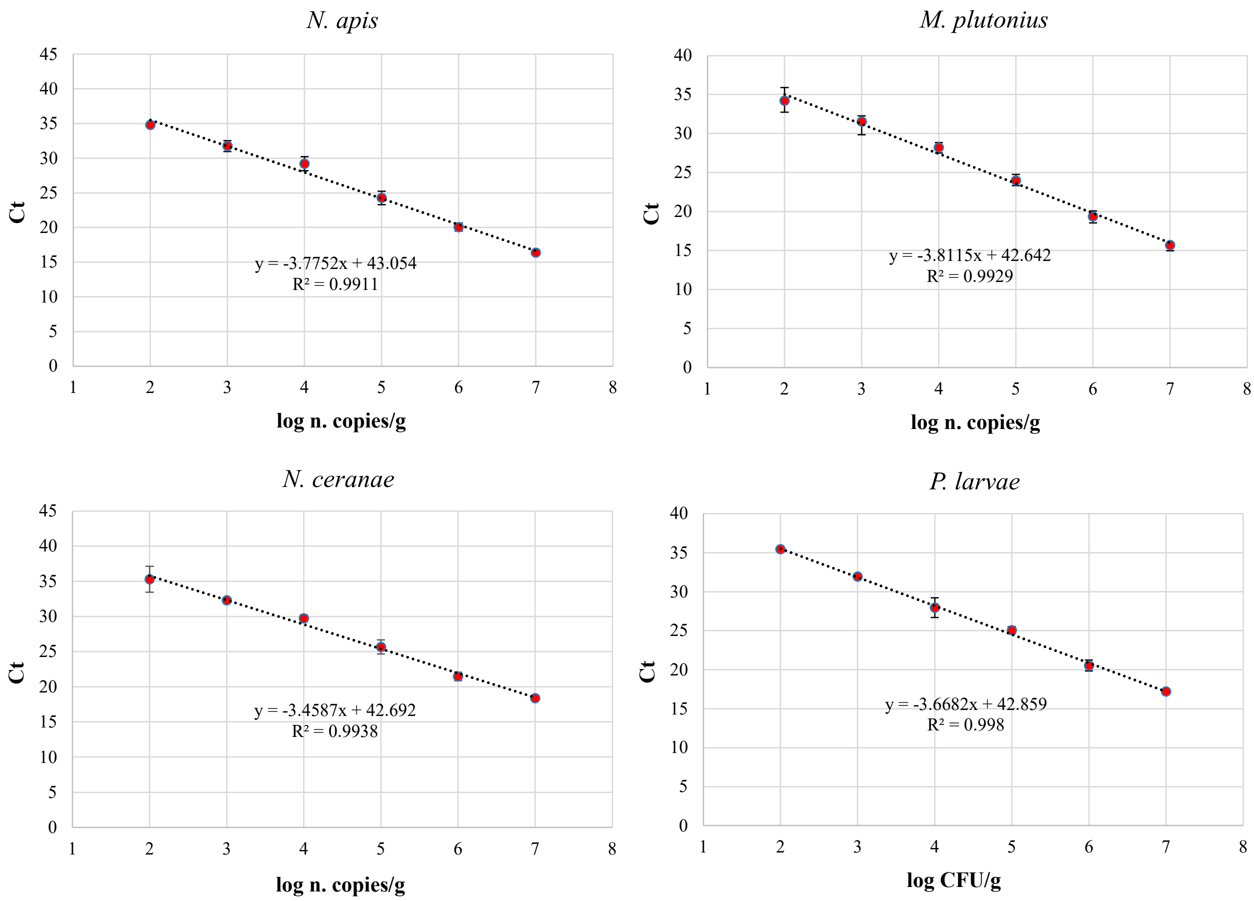

Figure S1. Calibration curves constructed in this study to quantify IPAs in honeybee samples with DNA extracted from honeybee samples negative for these pathogens and inoculated in three biological replicates with serial decimal dilutions of plasmid pUC57 containing the target region of *N. apis*, *N. ceranae*, *M. plutonius* or with known numbers of *P. larvae* cells using previously reported qPCR methods [17,23,24]. Only the linearity ranges are shown.
