# Supplementary material for: Distribution and Quantification of Infectious and Parasitic Agents in Managed Honeybees in Central Italy, the Republic of Kosovo, and Albania
Source: Microorganisms. 2026 Jan 17;14(1):219. doi: 10.3390/microorganisms14010219 (PMC12843687; doi:10.3390/microorganisms14010219)
Supplement: Supplementary file 1 [file microorganisms-14-00219-s001.zip › Table S1.pdf]

Table S1. IPAs co-infection profiles observed in honeybee samples from the Abruzzo and Molise regions (Italy), the Republic of Kosovo, and Albania in 2022 and 2023.

N. ceranae	***M. plutonius***	***P. larvae***	**ABPV**	**BQCV**	**CBPV**	**DWV-A**	**DWV-B**	**SBV**	***M. scalaris***	***S. tricuspis***
Italy 2022										
+									+	
+								+		
+				+						+
+				+	+		+			
+				+	+	+	+			
+				+	+	+		+	+	
+				+	+	+	+			+
+				+	+	+	+	+		+
+		+		+	+	+			+	
+		+		+	+	+	+	+	+	+
+			+	+	+	+	+	+		
+	+		+	+						
+	+		+		+	+			+	
+	+		+		+	+				+
+	+		+	+	+	+	+	+	+	+
+	+									
+	+									+
+	+	+		+	+	+		+	+	
+	+	+				+				
	+								+	
	+		+	+	+	+				
	+			+		+	+			
	+		+	+					+	
	+		+	+	+	+	+	+		
	+			+		+	+		+	
	+			+	+	+	+		+	
	+			+	+	+	+	+		+
	+	+		+	+	+	+	+		+
	+	+		+						
	+	+		+	+				+	
	+	+		+	+	+	+	+	+	+
	+	+		+	+	+		+	+	
	+	+		+				+	+	
	+	+		+	+	+	+	+	+	
	+	+		+	+	+	+	+		+
	+	+	+	+	+	+	+	+		
		+		+		+	+			
		+		+		+		+	+	
		+		+	+	+	+	+		
			+	+						+
			+	+	+	+	+	+		+
				+						
				+					+	
				+	+			+	+	
				+	+	+		+		
				+	+	+	+			+
				+	+	+	+	+		
				+		+	+		+	
				+		+				+
				+		+	+			
				+				+	+	
				+	+	+	+	+		
				+	+	+	+	+	+	+
					+	+	+	+		
						+			+	
						+	+			+

N. ceranae	***M. plutonius***	***P. larvae***	**ABPV**	**BQCV**	**CBPV**	**DWV-A**	**DWV-B**	**SBV**	***M. scalaris***	***S. tricuspis***
Italy 2023										
+				+	+				+	
+		+		+	+	+				
+				+	+	+	+	+		
+	+			+	+	+	+	+		+
	+		+	+		+	+			
	+			+		+				
	+			+	+	+	+	+		+
	+			+	+	+	+	+	+	+
	+			+	+					
	+			+	+	+		+		
	+			+						
	+	+		+	+	+				
	+	+		+	+	+	+	+		
	+	+		+	+	+	+		+	
	+	+		+	+	+	+	+	+	
	+	+	+	+		+	+		+	
	+	+		+	+	+	+	+	+	
		+		+	+	+	+	+		
		+		+	+			+		
		+		+						
		+		+		+		+		
		+	+	+		+		+		
		+		+	+		+		+	
		+		+	+			+	+	
		+		+	+	+		+	+	+
			+	+	+	+	+	+		

N. ceranae	*M. plutonius*	*P. larvae*	**ABPV**	**BQCV**	**CBPV**	**DWV-A**	**DWV-B**	**SBV**	*M. scalaris*	*S. tricuspis*	*N. ceranae*	*M. plutonius*	*P. larvae*	**ABPV**	**BQCV**	**CBPV**	**DWV-A**	**DWV-B**	**SBV**	*M. scalaris*	*S. tricuspis*
Albania 2022																					
+				+	+	+	+	+													
+		+		+	+	+	+	+													
+		+		+		+	+	+													
Republic of Kosovo 2022											Kosovo 2023										
+		+		+			+				+		+		+		+	+			
+		+		+			+			+	+		+		+		+	+	+		
+		+		+			+	+			+		+		+		+	+	+		+
+		+		+		+	+				+		+		+	+	+	+			
+		+		+		+	+		+		+		+		+	+	+	+	+		
+		+		+		+	+	+			+		+		+	+		+	+		
+		+		+		+	+	+	+		+		+		+			+	+		
+		+		+	+	+	+				+		+		+			+			
+		+		+	+	+	+	+			+		+		+						
+		+		+	+	+		+			+		+	+	+		+	+			
+		+		+		+		+			+		+	+	+		+	+	+		
+		+		+		+	+				+		+	+	+	+		+	+		
+		+			+	+	+				+		+	+	+	+	+	+	+		
+		+				+	+				+				+			+			
+		+					+				+				+			+	+		
+		+					+	+			+	+	+		+			+			
+		+			+			+	+				+		+			+			
+		+			+		+						+		+			+	+		
+		+		+	+		+	+					+		+		+	+			
+		+		+	+		+	+	+				+		+	+	+	+	+		
+		+		+	+		+						+		+		+		+		
+		+		+	+								+	+	+		+	+	+		
+		+		+				+													
+		+		+			+	+													
+		+		+			+														
+		+		+																	
+		+	+		+	+	+	+													
+		+	+	+		+	+	+													
+		+	+	+		+															
+		+	+	+		+	+														
+		+	+	+			+														
+			+	+	+																
+			+	+	+		+	+													
+			+	+		+															
+			+	+		+		+													
+			+	+	+	+	+	+													
+			+		+		+	+													
+			+	+	+			+													
+				+		+	+														
+				+		+	+		+												
+				+	+	+	+														
+						+	+														
+						+		+													
+					+		+	+	+												
+						+	+	+													
+				+	+		+	+													
+				+			+														
+				+				+	+												
+				+	+	+	+	+													
+				+		+															
+				+		+	+	+													
+	+			+	+		+	+													
+	+	+		+		+		+	+												
+	+	+		+		+	+	+	+												
+	+	+		+			+	+		+											
+	+	+	+	+			+														
+	+	+	+		+		+	+													
	+	+		+	+	+	+	+	+												
		+					+		+												
		+		+		+	+	+													
		+		+	+	+	+	+													

+	+	+	+	+		+
+	+	+		+		
	+	+		+	+	+
		+		+	+	+
		+			+	+
